# Telomerase expression and telomere length in breast cancer and their associations with adjuvant treatment and disease outcome

**DOI:** 10.1186/bcr2893

**Published:** 2011-06-06

**Authors:** Lingeng Lu, Chong Zhang, Gongjian Zhu, Melinda Irwin, Harvey Risch, Guido Menato, Marco Mitidieri, Dionyssios Katsaros, Herbert Yu

**Affiliations:** 1 Department of Epidemiology and Public Health, Yale Cancer Center, Yale University School of Medicine, 60 College Street, New Haven, CT 06520-8034, USA; 2 Gansu Provincial Hospital for the Protection of Mother and Baby's Health, 143 North Street, Lanzhou 730050, China; 3 Gansu Provincial Academy of Medical Science, Gansu Provincial Tumor Hospital, 2 Xiaoxihu East Street, Lanzhou 730050, China; 4 Department of Obstetrics and Gynecology, Gynecologic Oncology and Breast Cancer Unit, University of Turin, Via Ventimiglia 3, Turin 10126, Italy

## Abstract

**Introduction:**

Telomere length plays important roles in maintaining genome stability and regulating cell replication and death. Telomerase has functions not only to extend telomere length but also to repair DNA damage. Studies have shown that telomerase may increase cancer cell resistance to DNA-damaging anticancer agents; tamoxifen may suppress telomerase expression in breast cancer cells. This study aimed to investigate the role of telomere length and telomerase activity in breast cancer prognosis.

**Methods:**

qPCR and qRT-PCR were used to analyze telomere length and telomerase expression, respectively, in tumor samples of 348 breast cancer patients. Cox regression analysis was performed to examine telomere length and telomerase expression in association with disease-free survival and cause-specific mortality.

**Results:**

Telomere length had no relation to tumor features or disease outcomes. Telomerase expression was detected in 53% of tumors. Larger tumors or aggressive disease were more likely to have telomerase expression. Among patients treated with chemotherapy, high telomerase was found to be associated with increased risk of death (hazard ratio (HR) = 3.15; 95% CI: 1.34 to 7.40) and disease recurrence (HR = 2.04; 95% CI: 0.96 to 4.30) regardless of patient age, disease stage, tumor grade, histological type or hormone receptor status. Patients treated with endocrine therapy had different results regarding telomerase: high telomerase appeared to be associated with better survival outcomes. Telomerase expression made no survival difference in patients who received both chemotherapy and endocrine therapy.

**Conclusions:**

Overall, telomerase expression was not associated with disease outcome, but this finding may be masked by adjuvant treatment. Patients with high telomerase expression responded poorly to chemotherapy in terms of disease-free and overall survival, but fared better if treated with endocrine therapy.

## Introduction

Telomeres are repeated sequences of oligonucleotides (TTAGGG) located at the ends of chromosomes, and have important functions in regulating cell replication and maintaining genome integrity [1-3]. The length of telomeres decreases with the number of cell divisions; reduced telomere length carries functional cellular signals. Cells enter senescence or initiate apoptosis when telomere length is reduced to a critical level. Shortened telomere length often leads to genome instability, resulting in loss of cell-cycle control, a hallmark of cancer. Telomere shortening has been found to be associated with increased risk of several human cancers, including bladder, lung, esophagus, pancreas, ovary and thyroid [4-8].

Telomerase, a ribonucleoprotein complex composed of telomerase reverse transcriptase (TERT), telomerase RNA (TERC) and dyskerin [9], is a nuclear enzyme that increases the length of telomeres. Using TERC as a template, TERT adds telomeric repeats to the ends of chromosomes [10,11]. While it is important to maintain appropriate telomere length for genome stability, dysregulated telomere elongation also makes cells immortal, which is a feature of cancer cells. Telomerase expression is low during early carcinogenesis, but markedly increases in tumor invasion, preventing cancer cells from entering senescence or apoptosis [12-14]. Compared to that in adjacent normal tissues, the activity of telomerase is up-regulated in many types of cancer, including breast [15-17]. Several studies have shown high telomerase activity to be linked to poor prognosis of breast cancer [18-20]. Based on the experimental observations of *in vitro *cell cultures and *in vivo *animal models, telomerase inhibitors can reduce tumorigenicity and suppress breast cancer growth and metastasis [21-23]. Up-regulated telomerase activity and telomere elongation may also increase drug resistance in breast and colorectal cancer cell lines [17,24]. Telomerase inhibitors can increase cancer cell death when used in combination with DNA-damaging anticancer drugs [25]. However, our understanding of telomerase activity in breast cancer remains limited. The purpose of this study was to investigate the possible effects of telomerase expression and telomere length on breast cancer treatment outcomes.

## Materials and methods

### Study patients

Patients undergoing surgery for primary breast cancer between January 1998 and July 1999 in the University Hospital at University of Turin were recruited to a clinical follow-up study. The study was approved by the university's ethical review committee, and a written consent form was obtained from each study participant. In total, 348 consecutive patients were enrolled in the study, with the average age of patients at surgery being 57 years (range: 23 to 84). Of the patients, 302 were followed from surgery through February 2007. The median follow-up time was 86 months (range: 8 to 108). Clinical and pathological information on these patients was collected from the hospital records and pathology reports (Table 1). The majority of patients in the study were diagnosed with early stage cancer (36.4% stage I and 53.4% stage II). Patients with late stage diseases (stages III and IV) accounted for only 10.3%. Grades 1 (well differentiated), 2 and 3 tumors were seen in 16.6%, 41.1% and 42.3% of the patients, respectively. The major histological tumor type was ductal carcinoma (63.1%), followed by lobular carcinoma (16.1%). Other histological types were ductal-lobular or tubular-lobular mixed histologies which accounted for 10.7% and 10.1%, respectively. Two hundred one patients (58.1%) had tumors smaller than 2 cm, 120 patients (34.7%) had tumors between 2 cm and 5 cm, and 25 (7.2%) had tumors greater than 5 cm. One hundred sixty patients (46.8%) had lymph node-positive tumors. According to the 10% assay cutoff, 64.9% of the patients were ER positive, and 52.2% were PR positive.

**Table 1:** Clinical and pathological features of breast cancer patients in the study

Variable	Number	%
Stage (TMN) (n = 341)		
I	124	36.4
II	182	53.4
III	30	8.8
IV	5	1.5
Grade (n = 343)		
1	57	16.6
2	141	41.1
3	145	42.3
Histological type = 347)		
Ductal	219	63.1
Lobular	56	16.1
Mix	35	10.1
Other	37	10.7
Tumor size (n = 346)		
T1	201	58.1
T2	120	34.7
T3 and T4	25	7.2
Nodal status (n = 342)		
Negative	182	53.2
Positive	160	46.8
ER status (n = 342)		
Negative	120	35.1
Positive	222	64.9
PR status (n = 341)		
Negative	163	47.8
Positive	178	52.2
Treatment (n = 348)		
No adjuvant therapy	45	12.9
Chemotherapy	119	34.2
Endotherapy	77	22.1
Chemotherapy and endotherapy	107	30.8

The majority of patients (303 out of 348) received adjuvant therapy. Of these patients, 119 (34.2%) received chemotherapy, 77 (22.1%) had hormonal therapy, and 107 (30.8%) received both chemotherapy and endocrine therapy. Forty-five patients did not receive any adjuvant treatment. Chemotherapy protocols used for the patients included CMF (cyclophosfamide, methotrexate, 5 fluoro-uracil; dose: 600/60/600 mg/mq every three weeks for six cycles), CEF (cyclophosfamide, epirubicin, 5 fluoro-uracil; dose: 600/90/600 mg/mq every three weeks for six cycles), EPI-TAX (epirubicin-paclitaxel; dose: 90/175 mg/mq every three weeks for six cycles), EPI-VNB (epirubicin-vinorelbine; dose: 90/30 mg/mq every three weeks for six cycles), DTX-EPI-VNB (doxetaxel-epirubicin-vinorelbine; dose: 75/90/30 mg/mq every three weeks for six cycles), and TXT-EPI-VNB (paclitaxel-epirubicin-vinorelbine; dose: 175/90/30 mg/mq every three weeks for six cycles). Tamoxifen was the only endocrine therapy used at that time, and the dose was one 20 mg tablet per day for five years or until disease progression or unacceptable toxicity. By the end of follow-up, 55 of the 81 patients who had had recurrences had died. Among the 221 patients without disease progression, 5 had died.

### Tissue analysis for telomere length and telomerase expression

Fresh tumor samples were collected from the enrolled patients during surgery. The specimens were snap-frozen in liquid nitrogen immediately after resection and stored at -80°C until analysis. All tissue samples were examined by pathologists to confirm tumor content which varied between 80% and 90%. The specimens were pulverized manually in liquid nitrogen; aliquots of the tissue powders (about 100 mg each) were processed to extract DNA and total RNA separately, following conventional phenol-chloroform protocols. RNA samples were quantified and treated with DNase using a commercial kit (Ambion Inc, Austin, TX, USA).

A quantitative PCR (qPCR) developed by Cawthon [26] was used to analyze telomere length. The method compares the quantities of telomere and albumin in a common PCR reaction assuming that both products are amplified with similar efficiency. The primer sequences used were 5'- ACA CTA AGG TTT GGG TTT GGG TTT GGG TTT GGG TTA GTG T (forward) and 5'- TGT TAG GTA TCC CTA TCC CTA TCC CTA TCC CTA TCC CTA ACA (reverse) for telomere, and 5'-CGG CGG CGG GCG GCG CGG GCT GGG CGG AAA TGC TGC ACA GAA TCC TTG (forward) and 5'- GCC CGG CCC GCC GCG CCC GTC CCG CCG GAA AAG CAT GGT CGC CTG TT (reverse) for albumin. Chromo4™ continuous fluorescence detector system (MJ Research Inc., Waltham, MA, USA) was used to obtain Ct values for both telomere and albumin in each sample. Based on the formula 2^-(ΔCt)^, where ΔCt = Ct _telomere _- Ct _albumin_, the relative quantity of telomere versus albumin (T/S) was calculated. qPCR was done in 20 μl solution which mixed 10 μl of 2 × Quantifast SYBR green (Qiagen, Valencia, CA, USA) with telomere primers (900 nM), albumin primers (900 nM), and approximately 20 ng DNA template. The PCR conditions included initial denaturing at 95°C for 15 minutes, followed by 2 cycles of 15 sec at 94°C, and 15 sec at 49°C, and then 26 cycles of 15 sec at 94°C, 10 sec at 62°C, and 15 sec at 73°C with signal acquisition for telomere, 10 sec at 84°C, and 15 sec at 87°C with signal acquisition for albumin. After PCR amplification, melting curves were analyzed to confirm the size of PCR products. Each sample was run in duplicate. Samples with coefficient of variation >15% were reanalyzed.

Levels of telomerase expression along with an internal control (GAPDH) were determined with real-time reverse-transcriptase PCR (qRT-PCR). The forward and reverse PCR primers for telomerase described by Zhu *et al*. [27] were 5'- CGT CGA GCT GCT CAG GTC TT and 5'- AGT GCT GTC TGA TTC CAA TGC TT, respectively. The primer sequences for GAPDH and the qRT-PCR methods are described elsewhere [28-30]. Approximately 1.0 μg of DNase-treated total RNA from each sample was used for reverse transcription using the Cloned AMV First-Strand cDNA Synthesis kit (Invitrogen™, Carlsbad, CA, USA). qRT-PCR was performed using the Chromo4™ continuous fluorescence detector system. The PCR reaction was carried out in a 20 μl solution which contained 0.1 μg of cDNA template plus 10 μl of 2 × Power^®^ SYBR Green PCR Master Mix (Applied Biosystems, Foster City, CA, USA) and a pair of primers at a final concentration of 100 nM. PCR thermal cycle conditions included initial incubation at 50°C for 2 minutes, denaturing at 95°C for 10 minutes, and 40 cycles of 95°C for 15 sec and 60°C for 1 minute. Dissociation curves were generated after PCR to determine the size of PCR product. Each sample was tested in duplicate for each gene, and the analysis was repeated for those samples with large variation in their duplicates (coefficient of variation >5%).

### Statistical analysis

An expression index (EI) was calculated for telomerase expression using the formula 1,000 × 2 ^(-ΔCt)^, where ΔCt = Ct _(telomerase) _- Ct _(GAPDH)_. In data analysis, telomerase expression was analyzed as a categorical variable. Patients with undetectable telomerase, Ct _(telomerase) _>35, were grouped as low telomerase, and those with detectable telomerase as high telomerase. Telomere length was analyzed first as a continuous variable for its correlation with other variables, and then samples were classified into short and long telomere groups using median length as cutoff. The Chi-square test was used to compare differences in telomerase expression and telomere length among patients with different clinical and pathological features of breast cancer. Survival analysis was performed by proportional hazards regression methods to assess the associations of telomerase expression and telomere length with the risk of disease recurrence and death. SAS version 9.2 (SAS Institute, Cary, NC, USA) was used for all statistical analyses. *P-*values at 0.05 or smaller (two-sided) were deemed statistically significant.

## Results

### Telomerase expression and telomere length in breast cancer

Telomerase expression was analyzed in the 336 tumor samples that had sufficient quantities of RNA. Among these, 159 samples had low (undetectable) and 177 had high (detectable) telomerase expression. For the detectable samples, the average expression was 0.23 EI, and the expression range varied widely from 0.001 to 2,266 EI. The average value of telomere length in 348 tumor samples was 7.6, and the range was between 0.4 and 40.9. When analyzed as continuous variables, no correlation was found between telomerase expression and telomere length (r = 0.02, *P *= 0.746). This analysis was repeated for the samples with detectable expression (n = 177) using the log transformed data, and the result remained same. In categorical analysis, patients with detectable telomerase expression seemed more likely to have long telomeres compared to those with undetectable telomerase, but the difference was not significant (53.1% versus 45.3%, *P *= 0.152).

Telomerase expression and telomere length in relation to clinical and pathological features of breast cancer are shown in Table 2. A statistically significant association was observed between telomerase expression and tumor size (*P *= 0.035); patients with high telomerase expression tended to have larger tumors. Patients with high telomerase also seemed more likely to have stage III or IV disease (11.4% versus 9.1%) and grade 3 tumors (44.6% versus 38.8%), but these differences were not statistically significant. No significant associations were found between telomerase expression and histological type, lymph node involvement or hormone receptor status. Telomere length, either analyzed as a continuous or categorical variable, was not associated with any of the clinical or pathological features (categorical data in Table 2)

**Table 2:** Telomerase expression and telomere length in association with clinical and pathological features of breast cancer

Variable	Telomerase expression		P*	Telomere length		P*
	Low	High		Short	Long	
Telomere length (n = 336)			0.152			
Short	87 (54.7)	83 (46.9)				
Long	72 (45.3)	94 (53.1)				
Disease stage (n = 329)			0.487			0.788
I-II	140 (90.9)	155 (88.6)		149 (89.2)	146 (90.1)	
III-IV	14 (9.1)	20 (11.4)		18 (10.8)	16 (9.9)	
Tumor grade (n = 332)			0.292			0.300
1-2	96 (61.2)	97 (55.4)		93 (55.4)	100 (61.0)	
3	61 (38.8)	78 (44.6)		75 (44.6)	64 (39.0)	
Tumor size (n = 334)			**0.035**			0.648
T1	103 (65.6)	92 (52.0)		98 (58.3)	97 (58.4)	
T2	45 (28.7)	74 (41.8)		58 (34.5)	61 (36.8)	
T3 and T4	9 (5.7)	11 (6.2)		12 (7.1)	8 (4.8)	
Histological type (n = 335)			0.094			0.252
Ductal	98 (62.0)	114 (64.4)		101 (59.8)	111 (66.9)	
Lobular	31 (19.6)	25 (14.1)		35 (20.7)	21 (12.7)	
Mix	19 (12.0)	15 (8.5)		17 (9.5)	18 (10.8)	
Other	10 (6.4)	23 (13.0)		17 (10.1)	16 (9.6)	
Nodal status (n = 330)			0.618			0.191
Negative	79 (51.0)	94 (53.7)		94 (56.0)	79 (48.8)	
Positive	76 (49.0)	81 (46.3)		74 (44.0)	83 (51.2)	
ER status (n = 331)			0.782			0.884
Negative	53 (34.0)	62 (35.4)		59 (35.1)	56 (34..4)	
Positive	103 (66.0)	113 (64.6)		109 (64.9)	107 (65.6)	
PR status (n = 330)			0.352			0.181
Negative	70 (44.9)	87 (50.0)		86 (51.2)	71 (43.8)	
Positive	86 (55.1)	87 (50.0)		82 (48.8)	91 (56.2)	

### Telomerase expression and telomere length in relation to disease outcomes

Table 3 presents telomerase expression and telomere length in relation to disease outcomes. Patients with high telomerase expression had higher risk of disease recurrence and death than those with low expression, but the elevated risks were not statistically significant. Patients with long or short telomere lengths had quite similar survival outcomes, either for disease recurrence or death. Furthermore, these findings did not seem to change substantially after the major clinical and pathological features of breast cancer were adjusted in the analysis, including age at surgery, disease stage, tumor grade, histological type and receptor status.

**Table 3:** Survival outcomes in association with telomerase expression and telomere length in all patients

Variable	Overall Survival		Disease-free Survival	
	HR (95% CI)*	AHR (95% CI)**	HR (95% CI)*	AHR (95% CI)**
**Telomerase**				
Low telomerase	1.00	1.00	1.00	1.00
High telomerase	1.46 (0.87 to 2.45)	1.31 (0.78 to 2.22)	1.25 (0.80 to 1.95)	1.21 (0.79 to 1.94)
**Telomere length**				
Short telomere	1.00	1.00	1.00	1.00
Long telomere	0.79 (0.47 to 1.31)	0.83 (0.49 to 1.40)	0.84 (0.55 to 1.31)	0.83 (0.53 to 1.31)

*HR, hazard ratio; 95% CI, 95% confidence interval.

** AHR, adjusted hazard ratio; adjusted for patient age at surgery, disease stage, tumor grade, histological type, estrogen- and progesterone-receptor status.

However, since systemic adjuvant treatment may influence survival and interact with telomerase activity in its therapeutic effects, we performed survival analyses stratified by treatment modality (Table 4). Among patients treated with adjuvant chemotherapy, high telomerase expression was associated with higher risk of death and disease recurrence in comparison to low expression: adjusted hazard ratios 3.15 (95% CI: 1.34 to 7.40) and 2.04 (95% CI: 0.96 to 4.30), respectively. Interestingly, similar results were not observed in patients treated with endocrine therapy. These patients, in contrast, appeared to have better survival outcomes with high expression of telomerase, though none of the associations were statistically significant. For patients who received both chemotherapy and endocrine therapy, survival outcomes, either overall survival or disease-free survival, were similar according to high or low telomerase expression. No associations were found between telomere length and patient survival when patients were stratified by either adjuvant endocrine therapy or chemotherapy in both univariate and multivariate analyses (data not shown).

**Table 4:** Survival outcomes in association with telomerase expression stratified by adjuvant treatment

Variable	Overall survival		Disease-free survival	
	HR (95% CI)*	AHR (95% CI)**	HR (95% CI)*	AHR (95% CI)**
*Chemotherapy only (n = 119)*				
Low telomerase	1.00	1.00	1.00	1.00
High telomerase	2.05 (0.94 to 4.46)	3.15 (1.34 to 7.40)	1.32 (0.69 to 2.51)	2.04 (0.96 to 4.30)
*Endocrine therapy only (n = 77)*				
Low telomerase	1.00	1.00	1.00	1.00
High telomerase	0.65 (0.19 to 2.23)	0.43 (0.11 to 1.66)	0.92 (0.28 to 3.03)	0.78 (0.22 to 2.69)
*Chemo and endocrine therapy (n = 107)*				
Low telomerase	1.00	1.00	1.00	1.00
High telomerase	0.99 (0.37 to 2.63)	0.91 (0.33 to 2.55)	1.08 (0.52 to 2.23)	0.98 (0.46 to 2.09)

*HR, hazard ratio; 95% CI, 95% confidence interval.

** AHR, adjusted hazard ratio; adjusted for patient age at surgery, disease stage, tumor grade, histological type, estrogen- and progesterone-receptor status.

## Discussion

In this study, we analyzed breast tumors for telomere length and telomerase expression in order to assess their possible effects on disease outcomes. We found no evidence that telomere length was related to any of the major clinical or pathological features of the disease. In addition, we found no indication that telomere length was associated with patient survival outcomes, either disease-free survival or overall survival. We did notice, however, that tumors with longer telomere lengths were more likely to have detectable telomerase expression, although statistically significant correlation between the two was not observed. It has been reported that telomere length can be maintained without telomerase through an alternative mechanism, namely alternative lengthening of telomeres (ALT) [31]. Although the mechanisms by which ALT works remain unclear, it is thought that homologous DNA recombination is involved. About 10 to 15% of cancers including some with extremely poor prognosis use ALT to elongate telomere length [31]. Additionally, many environmental and host factors including psychological stress, physical activity, oxidative stress, hormones and growth factors have been shown to affect telomere length [32-36].

In this study, telomerase expression was found more often in larger tumors, and possibly in more aggressive disease. Telomerase expression was not associated with disease outcomes among patients when adjuvant treatment was not considered. However, if the treatment was considered, the associations between survival and telomerase became quite different. Patients who received adjuvant chemotherapy had worse survival outcomes if they had high telomerase expression in their tumors, but patients treated with endocrine therapy appeared to have better outcomes if their telomerase was high in the tumors. This discrepancy suggests that telomerase may interfere with adjuvant treatment. It is known that telomerase can maintain genome stability by elongating telomeres in normal cells, but evidence also suggests that telomerase may play different roles in tumors, and the effects are independent of telomere length. The enzyme may facilitate malignant transformation and render cells more resistant to apoptosis [37-39]. It is found that when telomerase is suppressed, short-term telomere integrity is not affected, but the overall chromatin configuration undergoes significant changes in terms of histone modification. These epigenetic alterations can impair DNA repair capacity and increase cell sensitivity to radiation [40]. Telomerase not only promotes DNA damage repair and facilitates cell survival, but also induces the resistance of cancer cells to anticancer agents that target DNA damage [25]. Our finding of unfavorable survival outcomes in association with high telomerase in patients treated with chemotherapy, but not in those treated with endocrine therapy, may be explained by the possibility that telomerase offsets the effect of chemotherapy since most chemotherapeutic drugs target DNA damage to induce cell death. In contrast, endocrine treatment exerts its effect through a mechanism that is completely different from chemotherapy.

If our observations are confirmed, our findings may have clinical implications in selecting adjuvant treatment for breast cancer. Patients with high or detectable telomerase expression may not be suitable for chemotherapy which targets DNA damage. Adjuvant treatment which combines chemotherapy with endocrine treatment may not be appropriate either because the treatment effects may cancel each other out. In our study, we observed no difference in survival benefit in this treatment subgroup between those with high and low telomerase expression. Thus, for patients with high telomerase, endocrine therapy may be the choice of adjuvant treatment. Our speculation that endocrine therapy may be a better choice for breast cancer patients with high telomerase activity is supported by laboratory finding that endocrine therapy antagonizes the activity of telomerase in breast cancer. A number of *in vitro *experiments have shown that both tamoxifen and raloxifene can inhibit telomerase expression in several breast cancer cell lines [41-43]. The inhibition of telomerase expression by tamoxifen is dependent on the presence of estrogen receptors [41]. Although our study shows a potential value of telomerase expression in breast cancer adjuvant treatment, these are preliminary findings which need to be confirmed by larger studies with consideration of molecular subtypes and endocrine therapy involving regimens other than tamoxifen.

Studies have shown that telomerase is highly active in most types of human cancers including breast cancer, but remains inactive in adjacent normal tissues. Telomerase inhibitors can suppress the growth and proliferation of tumor cells [17,44,45]. In our study, we found that telomerase expression was detectable in 53% of the tumors. This level of detection appears to be low when compared to some studies. Elkak *et al*. found that telomerase expression was detectable in all 116 breast cancer samples examined [18]. Other studies showed that 74% of breast carcinomas had detectable telomerase [46-48]. Using immunohistological staining, two studies found 72 to 86% of breast cancer samples having detectable telomerase activity [49,50]. However, there were two studies which found the frequency of detectable telomerase very similar to our study [51,52]. Reasons for the inconsistent detection of telomerase expression could be manifold, including different laboratory methods used for analysis and various cell contents in tissue samples. Liu and colleagues reported that normal mammary cells expressed higher telomerase than cancer cells [53]. Our findings of no associations between telomerase expression and disease stage or tumor grade were in agreement with those reported by Salhab and colleagues, who also found no association of telomerase with disease stage and tumor grade, although patients with poorly differentiated tumors (grade 3) appeared to have higher expression [19].

## Conclusions

In summary, we analyzed telomerase expression and telomere length in breast cancer and examined the relationships with disease outcomes. The study showed that telomere length was not associated with breast cancer clinical features or patient survival. Telomerase expression appeared to be slightly higher in tumors with longer telomeres as well as in larger tumors or aggressive disease. Overall, telomerase expression was not associated with disease outcome, but this finding may be masked by adjuvant treatment. Patients with high telomerase expression responded poorly to chemotherapy in terms of disease-free and overall survival, but fared better if treated with endocrine therapy. High or low telomerase expression made no difference in survival outcomes when patients received both chemotherapy and endocrine therapy. These findings suggest that telomerase activity may be a useful factor in determining the choice of adjuvant treatment for breast cancer patients.

## Abbreviations

AHR: adjusted Hazard Ratio; ALT: alternative lengthening of telomeres; CEF: cyclophosfamide, epirubicin, 5 fluoro-uracil; CI: confidential interval; CMF: cyclophosfamide, methotrexate, 5 fluoro-uracil; DTX-EPI-VNB: doxetaxel-epirubicin-vinorelbine; EPI-TAX: epirubicin-paclitaxel; EPI-VNB: epirubicin-vinorelbine; ER: estrogen receptor; GAPDH: glyceraldehyde 3-phosphate dehydrogenase; HR: hazard ratio; PR: progesterone receptor; qPCR: quantitative Polymerase Chain Reaction; qRT-PCR: quantitative reverse-transcription PCR; TERT: telomerease reverse transcriptase; TERC: telomerase RNA; TXT-EPI-VNB: paclitaxel-epirubicin-vinorelbine.

## Competing interests

The authors declare that they have no competing interests.

## Authors' contributions

LL, MI, HR, DK and HY participated in the conception and design of the study. LL, CZ, GZ, GM and MM acquired the data. LL, CZ, GZ, MI, HR, DK and HY analyzed and interpreted the data. LL, CZ, GZ, MI, HR, GM, MM, DK and HY prepared and revised the manuscript. All authors read and approved the final version of the manuscript.
